# Accuracy evaluation of mainstream and sidestream end-tidal carbon dioxide monitoring during noninvasive ventilation: a randomized crossover trial (MASCAT-NIV trial)

**DOI:** 10.1186/s40560-022-00603-w

**Published:** 2022-03-18

**Authors:** Masaaki Sakuraya, Eri Douno, Wakana Iwata, Akihiro Takaba, Kosuke Hadama, Natsuki Kawamura, Toshinori Maezawa, Kei Iwamoto, Yuya Yoshino, Kenichi Yoshida

**Affiliations:** 1 Department of Emergency and Intensive Care Medicine, JA Hiroshima General Hospital, Jigozen 1-3-3, Hatsukaichi, Hiroshima 738-8503 Japan; 2 Department of Emergency and Critical Care Medicine, Urasoe General Hospital, Okinawa, Japan; 3 Critical Care Medical Center, Hiroshima Prefectural Hospital, Hiroshima, Japan; 4 Emergency and Critical Care Center, Kurashiki Central Hospital, Okayama, Japan

**Keywords:** Blood gas analysis, End-tidal partial pressure of carbon dioxide, Mainstream capnography, Noninvasive ventilation, Partial pressure of carbon dioxide, Post-extubation

## Abstract

**Background:**

The end-tidal partial pressure of carbon dioxide (P_ET_CO_2_) can be used to estimate the arterial partial pressure of carbon dioxide (PaCO_2_) in patients who undergo mechanical ventilation via endotracheal intubation. However, no reliable method for measuring P_ET_CO_2_ during noninvasive ventilation (NIV) has been established. The purpose of this study was to evaluate the correlation and agreement between PaCO_2_ and P_ET_CO_2_ measured by these two methods and to compare them in patients who underwent NIV after extubation.

**Methods:**

This study was a randomized, open-label, crossover trial in a mixed intensive care unit. We included patients who were planned for NIV after extubation and for whom the difference between P_ET_CO_2_ and PaCO_2_ was ≤ 5 mmHg. We compared mainstream capnography using an inner cup via face mask (the novel method) with sidestream capnography (the previous method) during NIV. The relationships between PaCO_2_ and P_ET_CO_2_ were evaluated by computing the Pearson correlation coefficient, and the agreement between PaCO_2_ and P_ET_CO_2_ was estimated using the Bland–Altman method.

**Results:**

From April 2020 to October 2021, 60 patients were included to the study. PaCO_2_ and P_ET_CO_2_ were well correlated in both methods (the novel methods: *r* = 0.92, *P* < 0.001; the previous method: *r* = 0.79, *P* < 0.001). Mean bias between PaCO_2_ and P_ET_CO_2_ measured using the novel method was 2.70 (95% confidence interval [CI], 2.15–3.26) mmHg with 95% limits of agreement (LoA) ranging from − 1.61 to 7.02 mmHg, similar to the result of measurement during SBT (mean bias, 2.51; 95% CI, 2.00–3.02; 95% LoA, − 1.45 to 6.47 mmHg). In contrast, measurement using the previous method demonstrated a larger difference (mean bias, 6.22; 95% CI, 5.22–7.23; 95% LoA, − 1.54 to 13.99 mmHg).

**Conclusion:**

The current study demonstrated that the novel P_ET_CO_2_ measurement was superior to the previous method for PaCO_2_ prediction. During NIV, the novel method may collect as sufficient exhalation sample as during intubation. Continuous P_ET_CO_2_ measurement combined with peripheral oxygen saturation monitoring is expected to be useful for early recognition of respiratory failure among high-risk patients after extubation.

*Trial registration* UMIN-CTR UMIN000039459. Registered February 11, 2020.

**Supplementary Information:**

The online version contains supplementary material available at 10.1186/s40560-022-00603-w.

## Background

Among patients who undergo planned extubation, approximately 10%–20% are reintubated within 72 h, and most of them within 48 h [1–4]. Compared with successfully extubated patients, patients who are reintubated because of post-extubation respiratory failure might be at risk of worsening organ function [5]. Furthermore, reintubation is associated with a longer duration of stay in the intensive care unit (ICU) and hospital [6]. Noninvasive ventilation (NIV) is recommended to prevent post-extubation respiratory failure in high-risk patients [7]. In patients with acute respiratory failure who undergo NIV, delayed intubation increases mortality [8–10]. Therefore, careful respiratory monitoring is required to prevent delayed intubation.

The arterial partial pressure of carbon dioxide (PaCO_2_) should be maintained within an appropriate range during mechanical ventilation. PaCO_2_ measurements require arterial blood gas samples and are provided as intermittent information. The end-tidal partial pressure of carbon dioxide (P_ET_CO_2_), which is a continuous monitoring method, can be used to estimate PaCO_2_ in patients who undergo mechanical ventilation via endotracheal intubation. However, no reliable method for measuring P_ET_CO_2_ during NIV has been established. Although sidestream P_ET_CO_2_ measurements for NIV patients are moderately correlated with PaCO_2_, the 95% limits of agreement (LoA) are too large to be used in clinical settings [11, 12]. A possible explanation for the poor agreement between PaCO_2_ and P_ET_CO_2_ is the difficulty in collecting sufficient exhalation during NIV because of intentional leakage meant to avoid rebreathing. The cap-ONE mask set ^®^ (Nihon Kohden, Tokyo, Japan) is a unique interface for collecting exhalation with inner cups via face mask and measure using mainstream techniques, and is expected to predict the level of PaCO_2_ more accurately. However, this novel method has not yet been evaluated in clinical settings.

We hypothesized that this novel technique would be more accurate than the previous method. The purpose of this study was to evaluate the correlation and agreement between PaCO_2_ and P_ET_CO_2_ measured by these two methods and to compare them in patients who underwent NIV after extubation.

## Methods

### Trial design and setting

This study was a randomized, open-label, crossover trial conducted in a mixed ICU at the JA Hiroshima General Hospital. The study protocol was approved by the ethics committee of the JA Hiroshima General Hospital. This study was performed in accordance with the ethical standards laid down in the Declaration of Helsinki [13] and was registered at the UMIN Clinical Trials Registry on February 11, 2020 (UMIN 000039459), and reported in accordance with the CONSORT statement [14]. Written informed consent was obtained from all the patients or their relatives.

### Participants

Patients receiving mechanical ventilation, who were considered at a high risk of post-extubation respiratory failure and planned for NIV after extubation, were screened. We included patients if the difference between P_ET_CO_2_ and PaCO_2_ was ≤ 5 mmHg during the spontaneous breathing trial (SBT) and if an arterial line was placed. Patients with GCS ≤ 8, inability to protect the airway, hemodynamic instability, severe hypoxemia, agitation, NIV intolerance, chronic obstructive pulmonary disease, diagnosed pulmonary embolism or suspected, severe anemia (Hb < 7.0 g/dL), and arterial blood gas sample not collected were excluded. Moreover, patients whose cases were judged too difficult to include for analyses by a physician and those who refused consent were excluded.

Patients were considered at a high risk of post-extubation respiratory failure based on the criteria from a previous study (Additional file 1: Appendix S1) [15]. Briefly, as follows: age > 65 years; heart failure as the primary indication for mechanical ventilation; high severity score; obese; weaning process > 24 h (difficult or prolonged weaning, Additional file 1: Appendix S2) [16], 2 or more comorbidities (Additional file 1: Appendix S3), and mechanical ventilation for more than 7 days. All SBTs were performed at the lowest level of positive end-expiratory pressure (PEEP) and pressure support (PS) set at 5 cm H_2_O for 30–60 min. Considering these risks before extubation, the decision to perform NIV was made by the treating physicians.

### Randomization

Enrolled patients were randomized in a 1:1 ratio to receive either the previous method or the novel method as the first measurement. Randomization was performed using a computer-generated randomization table (www.randomization.com). Allocation results were placed into numbered sealed opaque envelopes containing monitoring allocations. Once the patient provided written informed consent, the clinicians participating in the study opened the envelopes in order.

### P_ET_CO_2_ monitoring methods

We compared the following two methods of P_ET_CO_2_ monitoring (the previous method and the novel method) in the included patients during NIV. After collecting the arterial blood gas sample, we switched to another method. The highest P_ET_CO_2_ value within one minute of collection of the blood gas sample was recorded. For the primary outcome, we assessed the correlations and agreements between the P_ET_CO_2_ and PaCO_2_ measurements performed by both methods.

### Previous method: sidestream monitoring using nasal prong and oral scoop

The Smart Capnoline ® Plus (Oridion Medical 1987 Ltd., Jerusalem, Israel) is a nasal prong and oral scoop for use in non-intubated patients with the dual purpose of delivering oxygen and collecting exhalation from both the nose and mouth (Additional file 1: Appendix S4). The length of the cannula was approximately 255 cm, and the delay in CO_2_ measurement was approximately 240 ms. The patients were fitted with a face mask over the nasal prong.

### Novel method: mainstream monitoring using the NPPV cap ONE mask ®

**Fig. 1:**
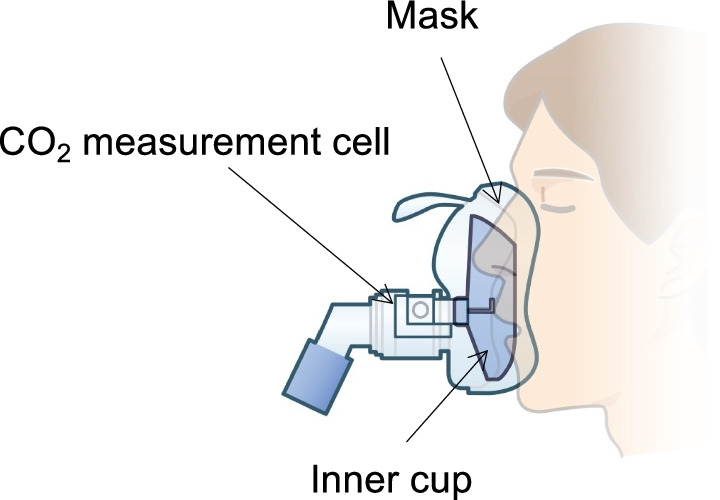
Mainstream monitoring using the novel method. An illustration of the NPPV cap ONE mask ® used in this study. The capnometer was calibrated in terms of the P_ET_CO_2_ reading before each use of the NKV 330 ventilator (Nihon Kohden Tokyo, Japan). In addition, the mainstream P_ET_CO_2_ sensor has a special anti-fog film on the specimen window, which guarantees accurate measurements for 72 h

The cap-ONE mask set ® (Nihon Kohden Tokyo, Japan) is a unique interface for collecting exhaled air samples using an inner cup in a face mask and assessing them using the mainstream techniques. The inner cup in the face mask was placed under the patient’s nose and over the mouth to guide the patient’s exhaled flow into the CO_2_ measurement cell (Fig. 1). The CO_2_ measurement cell was connected to the inner cup of the NPPV cap-ONE mask ®. The mainstream capnometer was designed to be placed on the CO_2_ measurement cell outside the mask. The capnometer was calibrated before each application of NIV.

### NIV for prevention of post-extubation respiratory failure

NIV was performed using the ventilator NKV 330 (Nihon Kohden Tokyo, Japan) and a face mask of the same size during both measurement periods. The NIV mode, setting, and duration were determined by treating physicians according to the following principles. It was recommended that the same mode and setting be maintained until the second measurement was completed, but they could be changed if necessary. NIV was continuously delivered immediately after extubation for a scheduled period to the next morning. NIV was interrupted once the patients were stable with oxygen administered via a mask or nasal cannula.

### Data collection

The following patient characteristics were recorded at admission: reason for ICU admission, age, sex, severity of illness (Acute Physiology and Chronic Health Evaluation II [APACHE II] score [17], Sequential Organ Failure Assessment [SOFA] score) [18], updated Charlson comorbidity index (CCI) [19], and nasal gastric tube placement. The following information was also recorded: NIV parameters (for example, mode, settings, tidal volume, minute ventilation, leakage), respiratory rate, blood pressure, heart rate, and peripheral oxygen saturation during each monitoring method. We performed blood gas analysis 30–60 min after each monitoring method session.

### Statistical analysis

We estimated the required sample size based on the correlation between P_ET_CO_2_ and PaCO_2_ values measured in previous studies conducted in non-intubated patients [12, 20–22]. A sample size of 60 measurements was required to achieve 90% power for detecting an effect size of 0.41 with α set at 0.05.

Data are expressed as mean with standard deviation (SD), medians with interquartile ranges (IQR), or numbers with corresponding percentages, as appropriate. Continuous variables were compared using the paired *t*-test or Wilcoxon signed-rank test, according to the data distribution. Dichotomous variables were analyzed using the Chi-square test or Fisher’s exact test. The relationships between PaCO_2_ and P_ET_CO_2_ were evaluated by computing the Pearson correlation coefficient, and the agreement between PaCO_2_ and P_ET_CO_2_ was estimated using the Bland–Altman method, in which bias was the mean difference between PaCO_2_ and P_ET_CO_2_, and the upper and lower LoA were the mean of the differences ± 1.96 SDs above and below the mean difference. Precision (the ability to reproduce the same measurement) was assessed based on the [bias—SD; bias + SD] interval, where SD is the SD of the distribution of the differences. Clinically unacceptable values were arbitrarily defined as values > 5 mmHg. In addition, we performed post hoc analyses to explore the source of the difference between PaCO_2_ and P_ET_CO_2_. The correlations and agreement between the P_ET_CO_2_ and PaCO_2_ measurements were evaluated in patients with small (≤ 40 L/min) and large (> 40 L/min) amounts of leakage. Furthermore, relationships between the difference and the following factors were evaluated using the Pearson correlation coefficient: the amount of leakage, tidal volume, respiratory rate, and minute ventilation. All statistical tests were two-sided, and a *p*-value < 0.05, indicating statistical significance. Statistical analyses were performed using Stata 15.1 (StataCorp LLC, College Station, TX, USA). The batplot command in Stata was used for the Bland–Altman analysis.

## Results

**Fig. 2:**
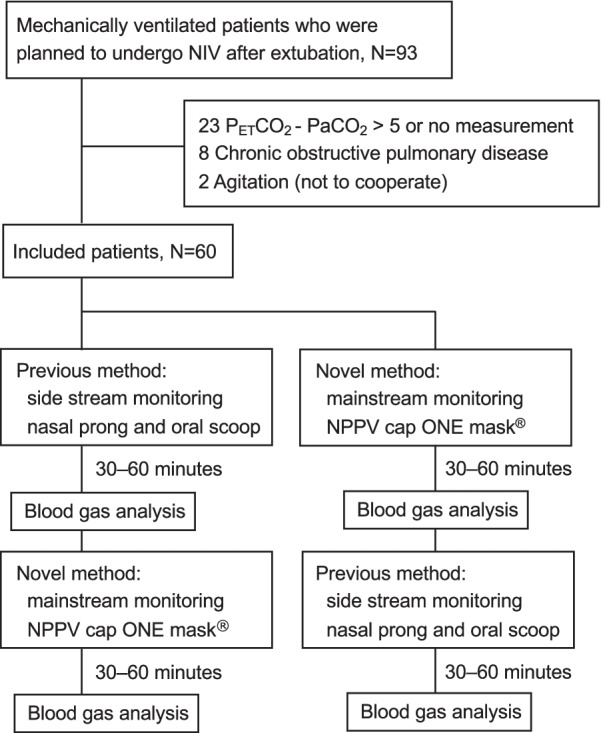
Patients flow diagram. P_ET_CO_2_, end-tidal partial pressure of carbon dioxide; NIV, noninvasive ventilation; PaCO_2_, arterial partial pressure of carbon dioxide

From April 2020 to October 2021, 326 adult patients were mechanically ventilated in the ICU. Of these patients, 93 patients who received NIV to prevent post-extubation respiratory failure were screened, and 60 patients were included in this analysis after inclusion and exclusion criteria were applied (Fig. 2). No patients were lost to follow-up during the study period.

Patient demographics and clinical characteristics are presented in Table 1 and Additional file 1: Table S1. Of the 60 patients, 37 (61.7%) were male, and the majority of patients were surgical patients (39 patients, 65.0%). The median (IQR) APACHE II score, SOFA score and updated CCI were 19 (range, 14–25), 8 (range, 7–11.5), and 3 (range, 2–4), respectively. The major comorbidities that the patient had were as follows: congestive heart failure (40 patients, 66.7%), renal disease (35 patients, 58.3%), diabetes with chronic complications (28 patients, 46.7%), and chronic respiratory failure (8 patients, 1.3%). The median duration of mechanical ventilation was 2 days (range, 2–4), and most patients were classified as having short weaning. Although all patients received NIV until the measurements using both monitoring methods were completed, three patients (5.0%) were reintubated during the ICU stay.

**Table 1:** Patient characteristics

	Included patients *N* = 60
Age, mean (SD), years	70.7 (11.2)
Male, n (%)	37 (61.7)
Body mass index, mean (SD), kg/m^2^	24.9 (4.0)
Patient category	
Non-scheduled surgery, n (%)	16 (26.7)
Scheduled surgery, n (%)	23 (38.3)
Medical, n (%)	21 (35.0)
APACHE II score, median (IQR), points*	19 (14–25)
SOFA score, median (IQR), points*	8 (7–11.5)
Updated Charlson comorbidity index, median (IQR), points	3 (2–4)
Chronic heart failure, n (%)	40 (66.7)
Chronic respiratory failure, n (%)	8 (1.3)
Renal disease, n (%)	35 (58.3)
Mechanical ventilation duration, days†	2 (2–4)
Weaning category^‡^	
Short weaning, n (%)	50 (83.3)
Difficult weaning, n (%)	10 (16.7)
Prolonged weaning, n (%)	0 (0)
Glasgow Coma Scale, median (IQR), points†	14 (13–15)
Vasoactive drugs use, n (%)^†^	32 (53.3)
Nasal gastric tube placement^†^, n (%)	40 (66.7)
Reintubation within ICU stay, n (%)	3 (5.0)
Length of ICU stay, days	4 (3–8)
Length of hospitalization^§^, days	33 (23.5–41)
ICU mortality, n (%)	0 (0)
Hospital mortality, n (%)	10 (16.7)

APACHE II, Acute Physiology and Chronic Health Evaluation II; ICU, Intensive Care Unit; SOFA, Sequential Organ Failure Assessment

^*^At ICU admission

^†^At the day of extubation

^‡^Defined by the WIND criteria

^§^Excluded 10 patients who died

The NIV settings and respiratory data are shown in Table 2. Although continuous positive airway pressure (CPAP) mode was used in most patients, only one patient underwent NIV in different modes in each section. The levels of PEEP and PS were 4 (range, 4–4) and 4 (range, 2–4) cmH_2_O, respectively. Although most of the respiratory statuses were not different between the two measurement periods, total leakage was smaller (35.0 [30.8–40.0] vs. 44.0 [38.3–52.2], P < 0.001), and the level of P_ET_CO_2_ was higher (35.5 [32–40] vs. 33 [27–36], P < 0.001) in patients using the novel method than those using the previous method, despite similar PaCO_2_ values.

**Table 2:** Ventilator settings, physiological data, and blood gas analysis in each section

	During SBT	Noninvasive ventilation		*P* value*
		Previous method	Novel method	
Ventilation mode				1.000
PSV, n (%)	NA	50 (83.3)	51 (85.0)	
CPAP, n (%)	NA	10 (16.7)	9 (15.0)	
F_I_O_2_, median (IQR)	0.3 (0.3–0.4)	0.3 (0.3–0.4)	0.3 (0.3–0.4)	1.000
PEEP, median (IQR), cmH_2_O	5 (5–5)	4 (4–4)	4 (4–4)	1.000
PS, median (IQR), cmH_2_O†	5 (5–5)	4 (2–4)	4 (2–4)	1.000
Heart rate, mean (SD), bpm	85.0 (15.5)	85.0 (14.7)	85.5 (15.1)	0.351
Mean blood pressure, mean (SD), mmHg	81.2 (11.6)	82.1 (11.6)	80.5 (11.6)	0.063
Respiratory Rate, mean (SD), breaths /min	15.9 (4.9)	18.2 (5.8)	19.1 (6.1)	0.108
Tidal Volume, mean (SD), mL	528 (157)	469 (130)	474 (122)	0.692
Minute ventilation, mean (SD), L/min	7.9 (2.3)	8.1 (3.2)	8.6 (3.3)	0.084
RSBI, mean (SD)	33.6 (16.1)	43.1 (20.4)	42.9 (17.0)	0.939
Total leakage, mean (SD), L/min	NA	45.1 (11.5)	36.1 (8.3)	< 0.001
PaO_2_, median (IQR), mmHg	87.3 (77.5–100.0)	86.2 (76.0–100.5)	89.6 (75.9–97.9)	0.793
P/F ratio, median (IQR)	290 (214–364)	271 (216–335)	280 (211–342)	0.601
PaCO_2_, mean (SD), mmHg	37.6 (5.7)	38.2 (5.5)	38.4 (5.4)	0.286
P_ET_CO_2_, mean (SD), mmHg	35.1 (5.8)	32.0 (6.3)	35.7 (5.5)	< 0.001
pH, mean (SD)	7.43 (0.05)	7.42 (0.05)	7.42 (0.05)	0.610

CPAP, continuous positive airway pressure; F_I_O_2_, fraction of inspiratory oxygen; NA, not applicable; PaCO_2_, arterial partial pressure of carbon dioxide; PaO_2_, arterial partial pressure of oxygen; PEEP, positive end expiratory pressure; P_ET_CO_2_, end-tidal partial pressure of carbon dioxide; P/F ratio, ratio of arterial oxygen partial pressure to fractional inspired oxygen; PS, pressure support; PSV, pressure support ventilation; RSBI, rapid shallow breathing index; SBT, spontaneous breathing trial

^*^Compared measurements in patients using the novel method with the previous method

^†^Among patients who underwent PSV during noninvasive ventilation

### ***Comparison between PaCO***_***2***_*** and P***_***ET***_***CO***_***2***_*** measurements***

**Fig. 3:**
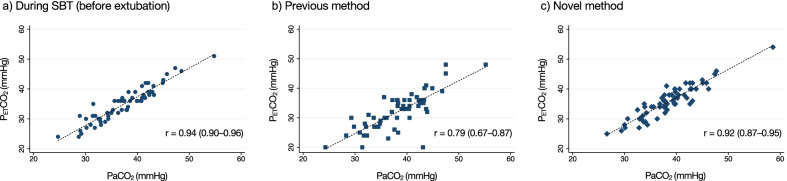
Correlations between PaCO_2_ and P_ET_CO_2_. a) During SBT (before extubation); b) the previous method; c) the novel method. There was a significant positive correlation between PaCO_2_ and P_ET_CO_2_ in all methods (during SBT *r* = 0.94 [95% CI, 0.90–0.96], *P* < 0.001; the previous method, *r* = 0.79 [95% CI, 0.67–0.87], *P* < 0.001; the novel method, *r* = 0.92 [95% CI, 0.87–0.95], *P* < 0.001). Abbreviations: CI, confidence interval; P_ET_CO_2_, end-tidal partial pressure of carbon dioxide; PaCO_2_, arterial partial pressure of carbon dioxide; SBT, spontaneous breathing trial

**Fig. 4:**
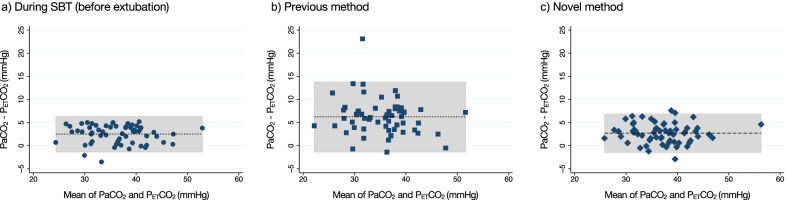
Bland–Altman plot of agreements between PaCO_2_ and P_ET_CO_2_. **a** During SBT (before extubation); **b** the previous method; **c**, the novel method. In each plot, bias is represented by the dashed line. The limits of agreement are represented by the gray zone. **a**, Bland–Altman analysis comparing PaCO_2_ and P_ET_CO_2_ for intubated patients during SBT. **b**, Bland–Altman analysis comparing PaCO_2_ and P_ET_CO_2_ for those who underwent noninvasive ventilation using the previous method (22 [36.7%] of these pairs had P_ET_CO_2_ values within 5 mm Hg of paired PaCO_2_). c), Bland–Altman analysis comparing PaCO_2_ and P_ET_CO_2_ for those who underwent noninvasive ventilation using the novel method (52 [86.7%] of these pairs had P_ET_CO_2_ values within 5 mm Hg of paired PaCO_2_). P_ET_CO_2_, end-tidal partial pressure of carbon dioxide; PaCO_2_, arterial partial pressure of carbon dioxide; SBT, spontaneous breathing trial

PaCO_2_ and P_ET_CO_2_ were well correlated in both methods (novel methods: *r* = 0.92, *P* < 0.001; previous method: *r* = 0.79, *P* < 0.001, Fig. 3). The results of the Bland–Altman analyses are shown in Fig. 4 and Table 3. Mean bias between PaCO_2_ and P_ET_CO_2_ measured using the novel method was 2.70 (95% CI, 2.15–3.26) mmHg with 95% LoA ranging from − 1.61 to 7.02 mmHg, similar to the result of measurement during SBT (mean bias, 2.51; 95% CI, 2.00–3.02; 95% LoA, − 1.45 to 6.47 mmHg). In contrast, measurement using the previous method demonstrated a larger difference (mean bias, 6.22; 95% CI, 5.22–7.23; 95% LoA, − 1.54 to 13.99 mmHg). The number of patients with ≤ 5 mmHg difference between PaCO_2_ and P_ET_CO_2_ was 52 (86.7%) using the novel method and 22 (36.7%) using the previous method.

**Table 3:** Results of Bland–Altman analysis of agreements between PaCO_2_ and P_ET_CO_2_

	Mean bias (95% CI)	95% Limits of agreement
During SBT, mmHg	2.51 (2.00–3.02)	− 1.45 to 6.47
Previous method, mmHg	6.22 (5.22–7.23)	− 1.54 to 13.99
Novel method, mmHg	2.70 (2.15–3.26)	− 1.61 to 7.02

CI, confidence interval; PaCO_2_, arterial partial pressure of carbon dioxide; P_ET_CO_2_, end-tidal partial pressure of carbon dioxide; SBT, spontaneous breathing trial

### Factor associated with the difference between PaCO_2_ and P_ET_CO_2_

For post hoc analyses, the correlations and agreement between the P_ET_CO_2_ and PaCO_2_ measurements were similar among subgroup patients stratified by the amount of leakage (Additional file 1: Figures S1 and S2). We also examined the Pearson correlation coefficient between the difference and respiratory status for each method. However, we found no factors that showed a good correlation with the differences (Additional file 1: Figure S3).

## Discussion

### Key findings

In the current study, among patients who underwent NIV to prevent post-extubation respiratory failure, both P_ET_CO_2_ monitoring methods demonstrated a good correlation with PaCO_2_. Compared with the measurement during SBT, mean bias using the novel method was similar, whereas it was larger in patients using the previous method. Furthermore, the difference between PaCO_2_ and P_ET_CO_2_ in most patients using the novel method was within an acceptable range.

### Relationship with previous studies

It has been challenging to estimate PaCO_2_ using P_ET_CO_2_ monitoring in patients undergoing NIV. Piquilloud et al. [11] evaluated P_ET_CO_2_ monitoring with the previous method among patients with hypercapnic respiratory failure, and it was not useful for predicting PaCO_2_ (mean bias, 14.7; 95% CI, 5.22–7.23; 95% LoA, − 6.6 to 36.1 mmHg). In a similar observational trial among patients with mixed respiratory failure conducted by Nouwen et al. [12], P_ET_CO_2_ monitoring showed good correlation but poor agreement for PaCO_2_. In our study, patients were excluded if the difference between P_ET_CO_2_ and PaCO_2_ was > 5 mmHg before extubation. Thus, most of the included patients were considered to have few physiological respiratory problems for P_ET_CO_2_ measurement (e.g., hemodynamic instability, ventilation perfusion mismatch, increased dead space, airflow limitation). The difference using the previous method was smaller compared with their studies, but still out of the acceptable range. The inaccuracy of the previous method might be due to insufficient sample collection, possibly because the sampling devices were small and the gap between the mask and skin created by nasal prong increased leakage. However, we assessed the correlations and agreements according to the amount of leakage via post hoc analysis, since larger amounts of leakage were observed among patients in whom the previous method was used. Our findings imply that the superiority of the novel method is not necessarily only to be ascribed to differences in the amount of leakage.

Mainstream and sidestream P_ET_CO_2_ measurements were not significantly different in estimating PaCO_2_ in mechanically ventilated patients [23, 24]. On the other hand, in an observational study evaluating both methods among non-intubated postoperative patients, the mainstream method was slightly more accurate than the sidestream method [21]. According to the results of another study among non-intubated patients in an emergency department, the mainstream method correlated but the sidestream method was poor, although both methods did not show good agreement for PaCO_2_ [20]. Therefore, the mainstream method was better at predicting the level of PaCO_2_ than the sidestream method in non-intubated patients because the sidestream method requires the collection of exhaled air samples using a sampling tube and the sampling gas may be diluted with air. In patients with NIV, high airflow, which flushes out air in an interface, may increase the air dilution of the exhalation sample. In our study, the novel method with mainstream capnography showed better correlation and agreement for PaCO_2_ than the previous method. Mainstream capnography may be more accurate in patients undergoing NIV. Another possible explanation is the difference in the sampling guides. The sampling guide of the previous method might be too small to collect sufficient exhalation. Therefore, it was unclear how much of a difference there would be between the mainstream and sidestream methods if a sufficient exhalation sample were obtained. Further evaluation is needed to clarify the superiority of the mainstream method given the same sampling system. Meanwhile, although an inner cup to collect exhalation samples in the novel method may increase rebreathing, the level of PaCO_2_ was not different between the two methods.

### Significance and implications

NIV is often used to prevent post-extubation respiratory failure and reintubation, which are associated with poor outcomes [5, 6]. Arterial blood gas analysis is recommended to assess patient respiratory status accurately and is evaluated more frequently in severe patients but not in continuous monitoring [26]. P_ET_CO_2_ monitoring, which has been used in intubated patients, is noninvasive and provides real-time information. Our findings imply that the novel method during NIV can collect enough exhaled samples during intubation. Since delayed intubation increases mortality [8–10], careful observation is needed to avoid intubation delays. Continuous P_ET_CO_2_ measurement combined with peripheral oxygen saturation monitoring is expected to be useful for the early recognition of respiratory failure and the prevention of delayed reintubation in patients who are at a high risk of post-extubation respiratory failure. Further study is needed to examine whether it improves clinical outcomes.

### Strengths and limitations

To the best of our knowledge, no previous research has demonstrated the usefulness of P_ET_CO_2_ monitoring during NIV. Our findings indicate that the results of previous studies were due to not only physiological issues, but also shortcomings of exhalation sample collection. Furthermore, the novel method using the cap-ONE mask set demonstrated good correlation and agreement with the level of PaCO_2_ in post-extubation patients with few physiological problems, compared with the previous method. However, this study has several limitations. First, patient respiratory status can affect the difference between PaCO_2_ and P_ET_CO_2_, which is expected to be larger with smaller tidal volume, higher respiratory rate, or higher airflow limitation [25]. In our post hoc analysis, none of the evaluated factors were associated with the difference between PaCO_2_ and P_ET_CO_2_, possibly because the level of PaCO_2_ was within normal ranges and respiratory status was stable in most of the included patients. Thus, our findings may have limited generalizability. For future investigation, it will be necessary to validate the novel method in patients with hypercapnic respiratory failure. Second, we measured the total amount of leakage without distinguishing between intentional and unintentional leakage. Unintentional leakage from the gap between the mask and the skin may be more closely associated with the collection of an exhalation sample than intentional leakage is. The relationship between collection of the exhalation sample and the different types of leakage should be investigated in more detail, as it has clinical implications. Third, we performed P_ET_CO_2_ measurements immediately after extubation. Ventilation and perfusion mismatch commonly increase immediately after extubation because of transient atelectasis. The difference between PaCO_2_ and P_ET_CO_2_ could change after extubation. Fourth, NIV indication was decided by the treating physicians, and no patient was intolerant to NIV. Consequently, the face mask could be appropriately fitted to collect exhalation in most patients. This was also a concern for the generalizability. Fifth, the novel method cannot be used with other NIV ventilators. The available opportunities for using this technology may be limited. For further clinical application, it must be made available for use with other NIV ventilators. Finally, the monitoring method could not be blinded, and this may have contributed to performance bias. Although it was not possible to blind data collectors, the highest P_ET_CO_2_ value within one minute of blood gas evaluation was measured to ensure objectivity.

## Conclusion

The current study demonstrated that the novel P_ET_CO_2_ measurement method was superior to the previous method for PaCO_2_ prediction. During NIV, the novel method may collect enough exhalation samples as during intubation. Continuous P_ET_CO_2_ measurement combined with peripheral oxygen saturation monitoring can be noninvasive and useful for early recognition of respiratory failure and to avoid delayed reintubation in patients who are at a high risk of post-extubation respiratory failure.

## Supplementary Information

**Additional file 1. Appendix S1.** Potential risks of post-extubation respiratory failure. **Appendix S2.** Weaning group according to the WIND criteria. **Appendix S3.** Updated Charlson comorbidity index. **Appendix S4.** The Smart Capnoline ® Plus. **Table S1.** Details of updated Charlson comorbidity index. **Fig. S1.** Correlations between PaCO_2_ and P_ET_CO_2_ according to leakage. **Fig. S2.** Bland–Altman plot of agreements between PaCO_2_ and P_ET_CO_2_ according to leakage. **Fig. S3**. Sensitivity analyses for correlations with difference (PaCO_2_—P_ET_CO_2_).

## Data Availability

The datasets used and analyzed during the current study are available from the corresponding author on reasonable request.

## Abbreviations

CI: Confidence interval
CPAP: Continuous positive airway pressure
ICU: Intensive care unit
IQR: Interquartile ranges
LoA: Limit of agreement
NIV: Noninvasive ventilation
PaCO_2_: Arterial partial pressure of carbon dioxide
PEEP: Positive end-expiratory pressure
P_ET_CO_2_: End-tidal partial pressure of carbon dioxide
PS: Pressure support
SBT: Spontaneous breathing trial
SD: Standard deviation
