# Supplementary material for: Accuracy evaluation of mainstream and sidestream end-tidal carbon dioxide monitoring during noninvasive ventilation: a randomized crossover trial (MASCAT-NIV trial)
Source: J Intensive Care. 2022 Mar 18;10:17. doi: 10.1186/s40560-022-00603-w (PMC8932153; doi:10.1186/s40560-022-00603-w)
Supplement: Supplementary file 1 — Additional file 1. Appendix S1. Potential risks of post-extubation respiratory failure. Appendix S2. Weaning group according to the WIND criteria. Appendix S3. Updated Charlson comorbidity index. Appendix S4. The Smart Capnoline ® Plus. Table S1. Details of updated Charlson comorbidity index. Fig. S1. Correlations between PaCO2 and PETCO2 according to leakage. Fig. S2. Bland–Altman plot of agreements between PaCO2 and PETCO2 according to leakage. Fig. S3. Sensitivity analyses for correlations with difference (PaCO2—PETCO2). [file 40560_2022_603_MOESM1_ESM.pdf]

Supplementary Online Content

Accuracy evaluation of mainstream and sidestream end-tidal carbon dioxide monitoring during noninvasive ventilation: a randomized crossover trial (MASCAT-NIV trial)

Table of Contents

Supplementary Methods

Appendix S1 Potential risks of post-extubation respiratory failure

.... 2

Appendix S2 Weaning group according to the WIND criteria

.... 3

Appendix S3 Updated charlson comorbidity index

.... 4

Appendix S4 The Smart Capnoline ® Plus

.... 5

Supplementary Results

Table

Table S1 Details of updated carlson comorbidity index

.... 6

Figure

Fig. S1 Correlations between $PaCO_2$ and $P_{ET}CO_2$ according to leakage

.... 7

Fig. S2 Bland-Altman plot of agreements between $PaCO_2$ and $P_{ET}CO_2$ according to leakage

.... 8

Fig. S3 Sensitivity analyses for correlations with difference ($PaCO_2$ - $P_{ET}CO_2$)

.... 9–10

Appendix S1 Potential risks of post-extubation respiratory failure [1]

Patients fulfilling at least 1 of the following criteria were considered at high risk of extubation failure:

- Age older than 65 years
- Heart failure as the primary indication for mechanical ventilation
- Moderate to severe chronic obstructive pulmonary disease
- Acute Physiology and Chronic Health Evaluation II score higher than 12 on extubation day
- Body mass index of more than 30 (calculated as weight in kilograms divided by height in meters squared)
- Airway patency problems
- Including high risk of developing laryngeal edema
- Inability to deal with respiratory secretions (inadequate cough reflex or suctioning >2 times within 8 hours before extubation)
- Difficult or prolonged weaning,
- A patient failing the first attempt at disconnection from mechanical ventilation
- Two or more items of charlson comorbidity index
- Mechanical ventilation for more than 7 days

Reference

1. Hernández G, Vaquero C, Colinas L, Cuena R, González P, Canabal A, et al. Effect of Postextubation High-Flow Nasal Cannula vs Noninvasive Ventilation on Reintubation and Postextubation Respiratory Failure in High-Risk Patients: A Randomized Clinical Trial. JAMA 2016;316(15):1565-1574.

Appendix S2 Weaning group according to the WIND criteria [1]

Group	Explanation
No weaning	Patients never experienced any separation attempt
Group 1 (Short weaning)	The first separation attempt resulted in a termination of the weaning process within 24 hours (successful separation or early death)
Group 2 (Difficult weaning)	Weaning was terminated after more than 1 day but in less than 1 week after the first separation attempt (successful separation or death)
Group 3 (Prolonged weaning)	Weaning was still not terminated 7 days after the first separation attempt (by success or death)

Reference

1. Béduneau G, Pham T, Schortgen F, Piquilloud L, Zogheib E, Jonas M, et al. Epidemiology of Weaning Outcome according to a New Definition. The WIND Study. Am J Respir Crit Care Med 2017;195(6):772-783.

Appendix S3 Updated carlson comorbidity index [1]

Item	Points
Congestive heart failure	2
Dementia	2
Chronic pulmonary disease	1
Rheumatologic disease	1
Mild liver disease	2
Moderate or severe liver disease	4
Diabetes with chronic complications	1
Hemiplegia or paraplegia	2
Renal disease	1
Any malignancy, including leukemia and lymphoma	2
Metastatic solid tumor	6
AIDS/HIV	4

Abbreviations: AIDS, acquired immunodeficiency syndrome; HIV, human immunodeficiency virus

Reference

1. Quan H, Li B, Couris CM, Fushimi K, Graham P, Hider P, et al. Updating and validating the Charlson comorbidity index and score for risk adjustment in hospital discharge abstracts using data from 6 countries. Am J Epidemiol 2011;173(6):676-682.

Appendix S4 The Smart Capnoline ® Plus

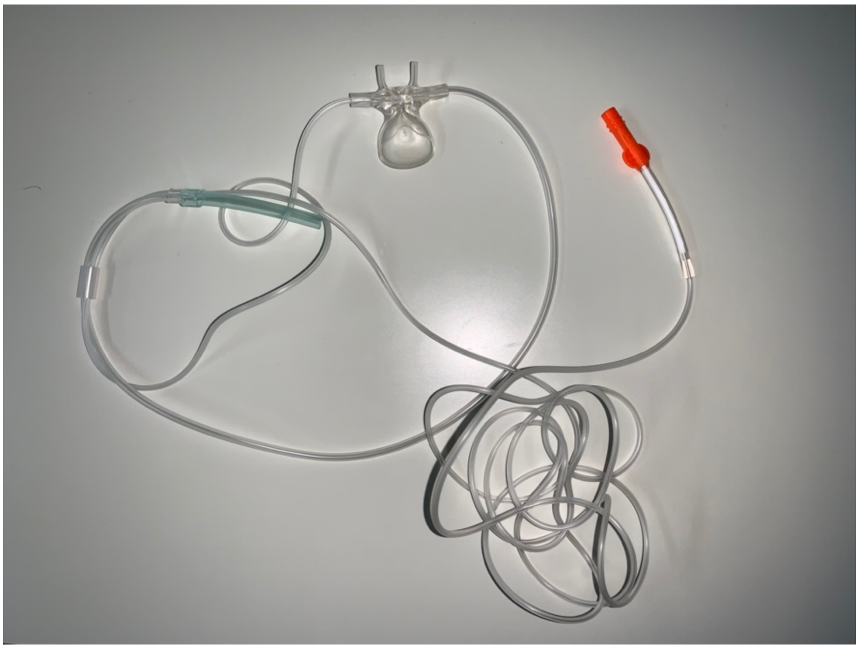

The Smart Capnoline ® Plus (Oridion Medical 1987 Ltd., Jerusalem, Israel) is a nasal prong and oral scoop for use in non-intubated patients with the dual purpose of delivering oxygen and collecting exhalation from both the nose and mouth. The length of the cannula was approximately 255 cm, and the delay in CO_2 measurement was approximately 240 ms. The patients were fitted with a face mask over the nasal prong.

Table S1 Details of updated carlson comorbidity index

Item	No. of the patients
Congestive heart failure, n (%)	40 (66.7)
Dementia, n (%)	3 (5.0)
Chronic pulmonary disease, n (%)	8 (1.3)
Rheumatologic disease, n (%)	0 (0)
Mild liver disease, n (%)	3 (5.0)
Moderate or severe liver disease, n (%)	2 (3.3)
Diabetes with chronic complications, n (%)	28 (46.7)
Hemiplegia or paraplegia, n (%)	2 (3.3)
Renal disease, n (%)	35 (58.3)
Any malignancy, including leukemia and lymphoma, n (%)	6 (10.0)
Metastatic solid tumor, n (%)	1 (1.7)
AIDS/HIV, n (%)	0 (0)

Fig. S1 Correlations between $PaCO_2$ and $P_{ET}CO_2$ according to leakage

1. Patients with small amount of leakage (total leakage < 40 L/min)

a) Previous method, N=22

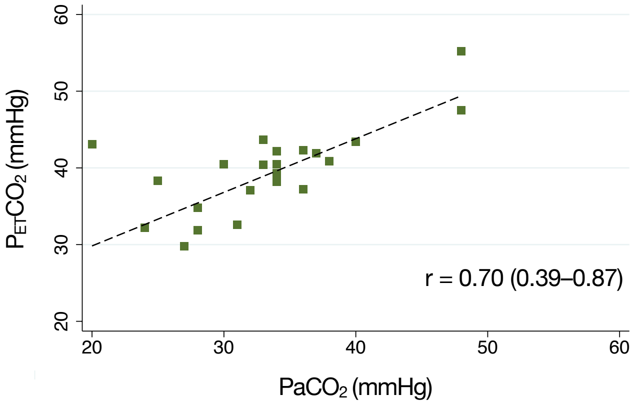

b) Novel method, N=46

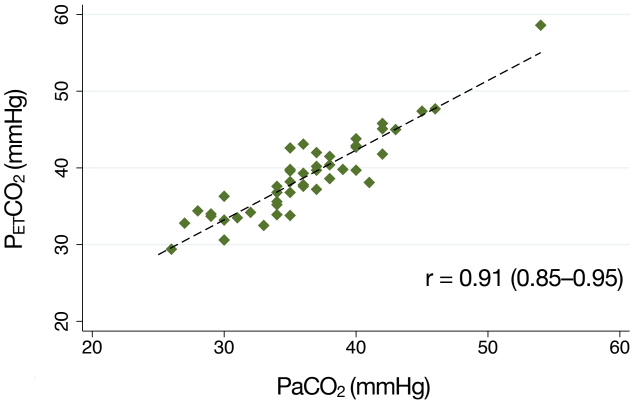

The previous method, r = 0.70 [95% CI, 0.39–0.87], P=0.002; the novel method, r = 0.91 [95% CI, 0.85–0.95], P <0.001)

2. Patients with large amount of leakage (total leakage > 40 L/min)

a) Previous method, N=38

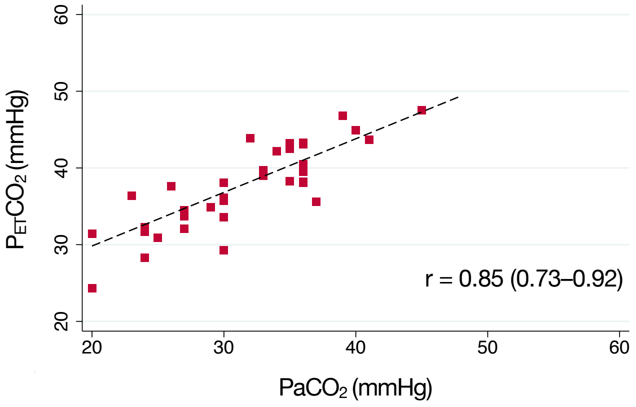

b) Novel method, N=14

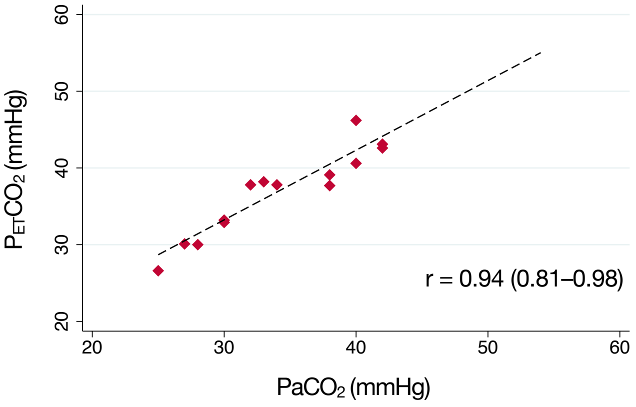

The previous method, r = 0.85 [95% CI, 0.73–0.92], P <0.001; the novel method, r = 0.94 [95% CI, 0.81–0.98], P <0.001)

Abbreviations: CI, confidence interval; $P_{ET}CO_2$, end-tidal partial pressure of carbon dioxide; $PaCO_2$, arterial partial pressure of carbon dioxide.

Fig. S2 Bland-Altman plot of agreements between $PaCO_2$ and $P_{ET}CO_2$among patients according to leakage

1. Patients with small amount of leakage (total leakage < 40 L/min)

a) Previous method, N=22 b) Novel method, N=46

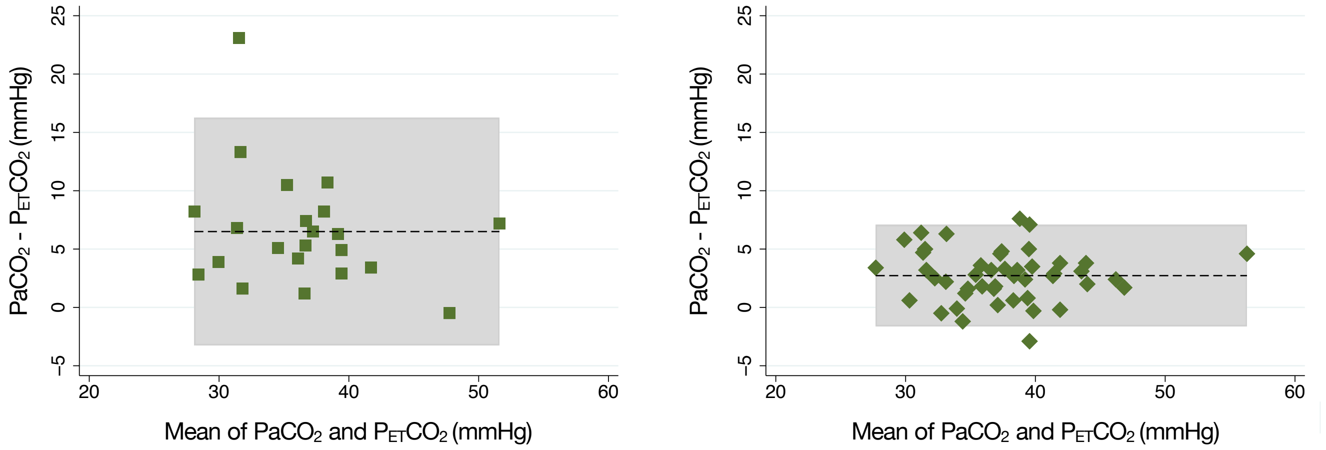

a) For Bland-Altman analysis in patients using the previous method. Mean bias between $PaCO_2$ and $P_{ET}CO_2$ measured using the previous method was 6.50 mmHg with 95% LoA ranging from -3.23 to 16.23 mmHg.

b) For Bland-Altman analysis in patients using the novel method. Mean bias between $PaCO_2$ and $P_{ET}CO_2$ measured using the novel method was 2.72 mmHg with 95% LoA ranging from -1.61 to 7.06 mmHg.

2. Patients with large amount of leakage (total leakage > 40 L/min)

a) Previous method, N=38 b) Novel method, N=14

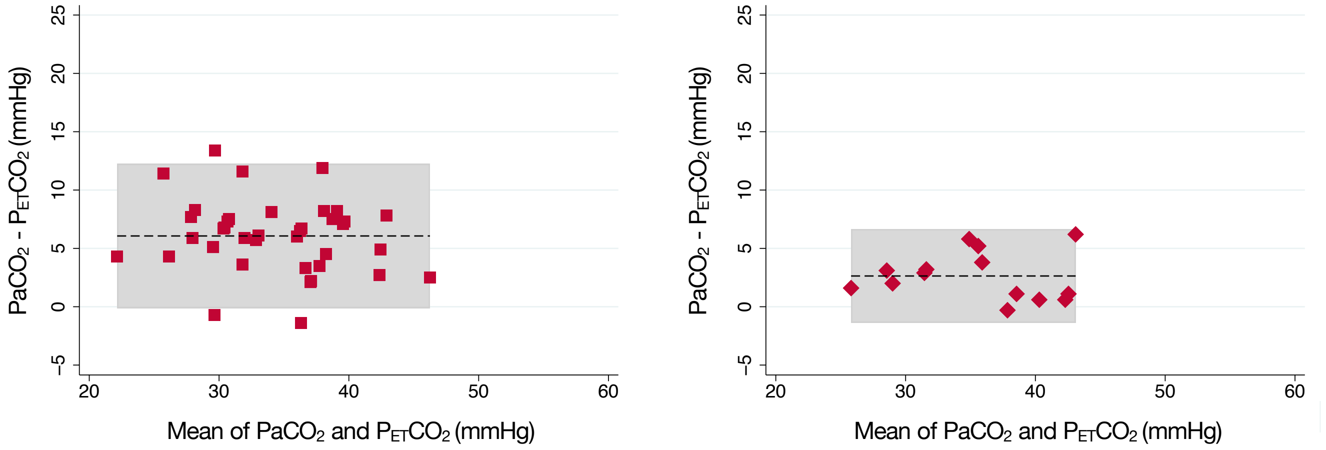

a) For Bland-Altman analysis in patients using the previous method. Mean bias between $PaCO_2$ and $P_{ET}CO_2$ measured using the previous method was 6.07 mmHg with 95% LoA ranging from -0.12 to 12.25 mmHg.

b) For Bland-Altman analysis in patients using the novel method. Mean bias between $PaCO_2$ and $P_{ET}CO_2$ measured using the novel method was 2.64 mmHg with 95% LoA ranging from -1.37 to 6.64 mmHg.

In each plot, bias is represented by the dashed line. The LoA is represented by the gray zone.

Abbreviations: LoA, limit of agreement; $P_{ET}CO_2$, end-tidal partial pressure of carbon dioxide; $PaCO_2$, arterial partial pressure of carbon dioxide; SBT, spontaneous breathing trial.

Fig. S3 Sensitivity analyses for correlations with difference (PaCO2 - $P_{ET}CO_2$)

1. Correlations between the difference ($PaCO_2$ - $P_{ET}CO_2$) and leakage

a) Previous method

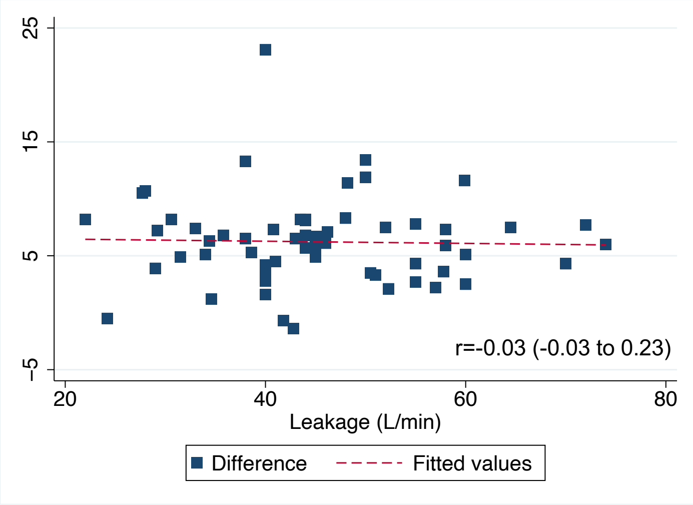

b) Novel method

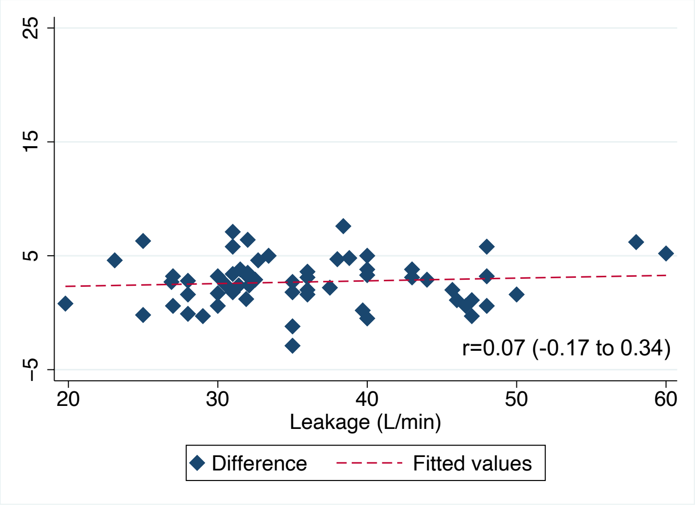

the previous method, r = -0.03 (95% CI, -0.03 to 0.23), P = 0.825; the novel method, r = 0.07 (95% CI, -0.17 to 0.34), P = 0.577.

2. Correlations between the difference ($PaCO_2$ - $P_{ET}CO_2$) and tidal volume

a) Previous method

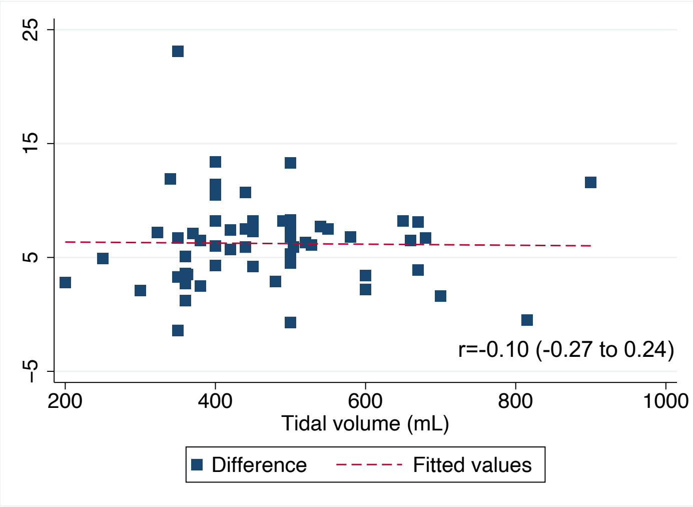

b) Novel method

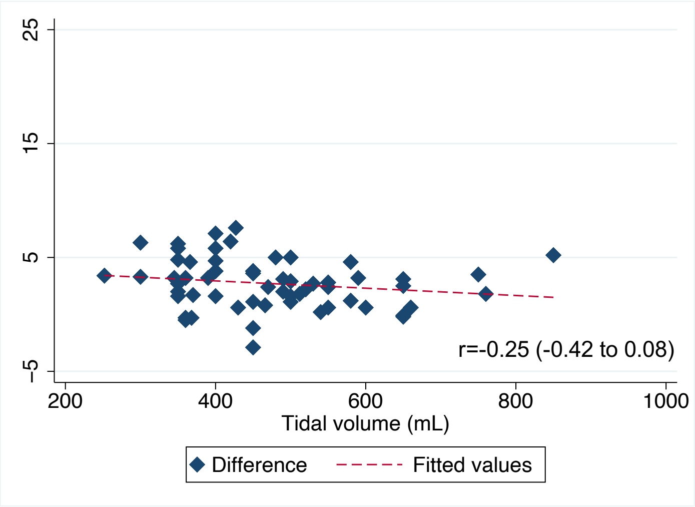

the previous method, r = -0.10 (95% CI, -0.27 to 0.24), P = 0.467; the novel method, r = -0.25 (95% CI, -0.42 to 0.08), P = 0.046.

3. Correlations between the difference ($PaCO_2$ - $P_{ET}CO_2$) and respiratory rate

a) Previous method b) Novel method

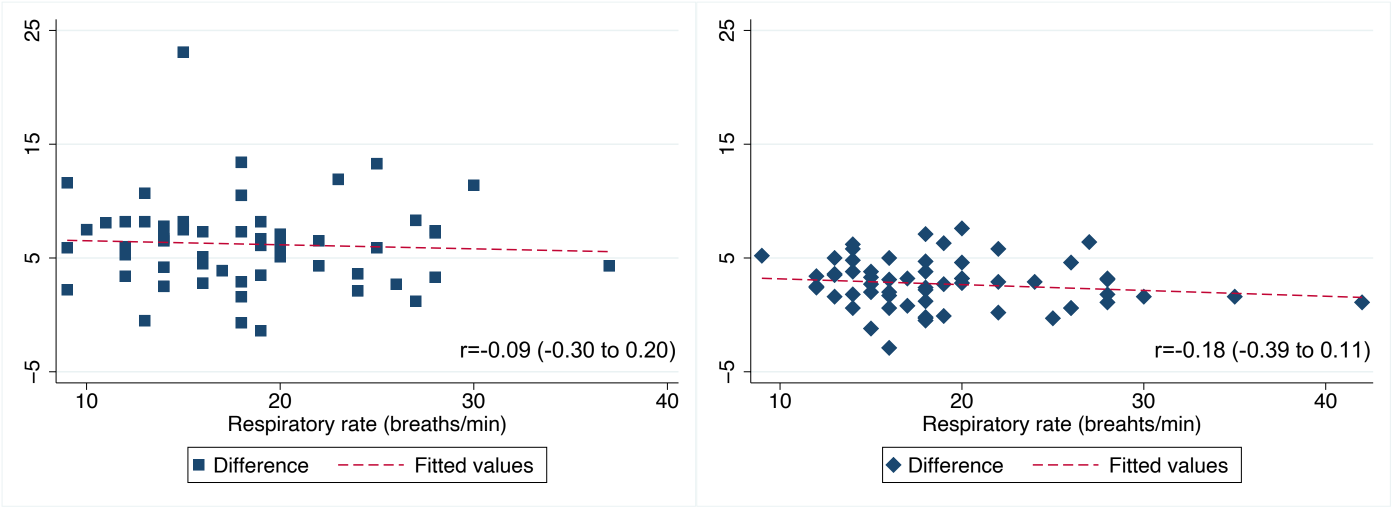

the previous method, r = -0.09 (95% CI, -0.30 to 0.20), P = 0.494; the novel method, r = -0.18 (95% CI, -0.39 to 0.11), P = 0.179.

4. Correlations between the difference ($PaCO_2$ - $P_{ET}CO_2$) and minute ventilation

a) Previous method b) Novel method

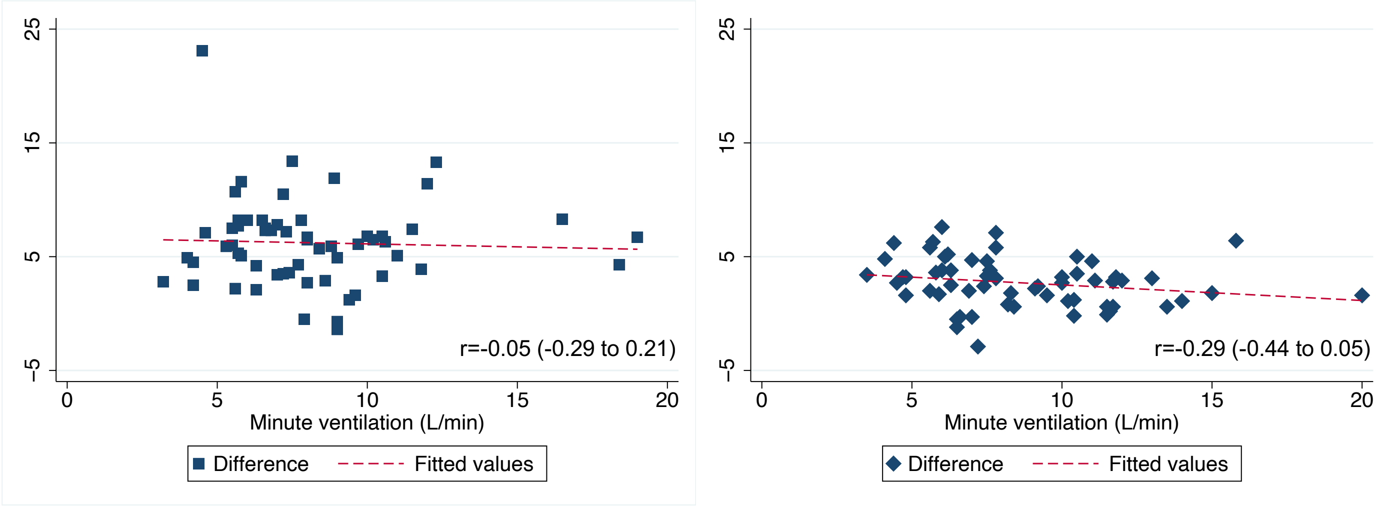

the previous method, r = -0.05 (95% CI, -0.29 to 0.21), P = 0.681; the novel method, r = -0.29 (95% CI, -0.44 to 0.05), P = 0.023.

Abbreviations: CI, confidence interval; $P_{ET}CO_2$, end-tidal partial pressure of carbon dioxide; $PaCO_2$, arterial partial pressure of carbon dioxide.
